# The importance of democratized resources in early-career training for bioimage analysts and bioimaging scientists

**DOI:** 10.3389/fbinf.2025.1693343

**Published:** 2025-10-30

**Authors:** Genevieve Laprade, Quinn Lee, Kristin L. Gallik, Michael Nelson, Natalie Woo, Celina Terán Ramírez, Alexis Ricardo Becerril Cuevas, Kevin W. Eliceiri, Corinne Esquibel

**Affiliations:** 1 BioImaging North America, Morgridge Institute of Research, Madison, WI, United States; 2 Department of Physiology, McGill University, Montreal, QC, Canada; 3 Center for Biomedical Research Support, University of Texas at Austin, Austin, TX, United States; 4 Optical Imaging Core, Van Andel Institute, Grand Rapids, MI, United States; 5 Department of Biomedical Engineering, University of Wisconsin-Madison, Madison, WI, United States; 6 Laboratorio Nacional de Microscopía Avanzada, Instituto de Biotecnología, Universidad Nacional Autónoma de México, Cuernavaca, Morelos, Mexico; 7 Hannover Medical School, Institute of Virology, Hanover, Germany; 8 Centre for Structural Systems Biology, Hamburg, Germany

**Keywords:** bioimaging networks, community, imaging scientists, microscopy, image analysis, early career, democratized resources

## Abstract

The fields of bioimaging and image analysis are rapidly expanding as new technologies transform biological questions into novel insights. While professionals of varying expertise are essential to achieving these advancements, early-career scientists—a prominent user group within the imaging community—are often assumed to have the prerequisite knowledge and ability to use these tools. This demographic, consisting of students, post-docs, and bioimage analysis trainees, is critical for the field to continue to evolve and flourish. However, obstacles such as geographic location, language barriers, insufficient funding or training, and instrument availability hinder access to resources and introduce significant knowledge gaps, especially for scientists in early-career stages. Democratized resources for bioimaging and analysis such as forums, community organizations, and publicly available datasets have been helpful in overcoming barriers to access for early-career scientists. Here, we discuss the current tools and resources available for early-career researchers, highlight their limitations from the learners’ perspective, and propose strategies to better support this group. As bioimage analysis extends broadly into many scientific disciplines, we implore all members of this community, regardless of experience level, to empower next-generation scientists.

## Graphical Abstract

**Figure:**
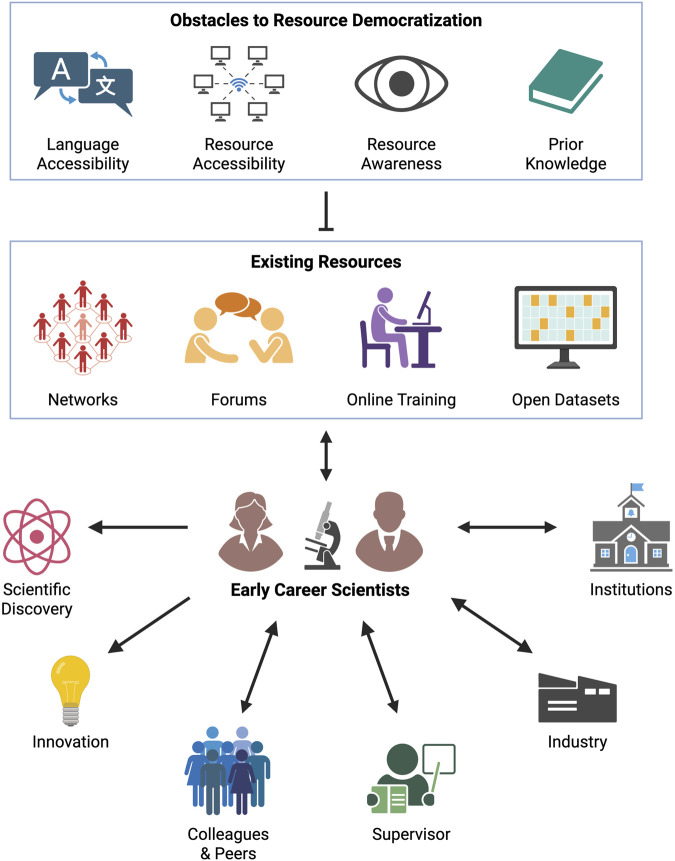
Democratized tools such as bioimaging networks, virtual forums, online training material, and open datasets improve the training of early-career scientists. These tools help promote scientific discoveries and innovation to benefit colleagues, supervisors, academic institutions, and industry. In turn, trainees and early-career scientists can improve and develop democratized tools. This progress is challenged by limitations in language accessibility, resource availability and awareness, and prior knowledge. Created in BioRender. Laprade, G. (2025).

## Introduction

Bioimaging is a dynamic field that is vital to answering questions across life science disciplines. Many elements are involved in properly designing and conducting a bioimaging experiment, including sample preparation, equipment use, image analysis, and data management. This often requires a certain degree of funding, expertise, and technology that may not be readily available in a lab. Moreover, the emergence of bioimage analysis as a discipline further underscores the requisite knowledge needed for research success (Cimini et al., 2024).

Many institutions have core facilities to support their local scientists, which house experts in various fields of scientific research such as microscopy and image analysis. Core personnel help circumvent common pitfalls such as missing appropriate controls, incorrect selection of fluorescent probes, and inapt imaging parameters. Thus, utilizing core facility services and applying their expert knowledge may lead to better research and publications. Scientists should be encouraged to reach out to their institute’s imaging core facility for guidance. However, not all institutions have this type of shared research infrastructure. For early-career scientists, a demographic composed of students, post-docs, and other junior researchers, the resources available in the lab and institutional environment can have a significant impact on their ability to learn and, by extension, on their career trajectories and the vitality of bioimaging as a field. Digital access to imaging tools is a powerful way to bypass these limitations and to disseminate microscopy and image informatics practices.

Community resources, including both networks of expertise such as bioimaging societies and tangible assets such as online training materials and publicly accessible datasets, provide training opportunities to overcome gaps in professional development. We believe the maximal impact of these tools requires that they be democratized and made as accessible as possible for all scientists. This requires that they be free or low-cost and readily available. Digital access to these resources provides a powerful means of democratizing microscopy training and extending early-career education beyond the physical instrument.

As the global scientific community becomes increasingly interconnected, easy-to-use online tools offer a unique opportunity to address individuals’ knowledge gaps. Democratizing training material by increasing its accessibility to all trainees helps enhance learning, which in turn produces better researchers and experimental outcomes. Importantly, democratized resources encourage innovative science by exposing early-career individuals to new ideas and groups beyond their local environment. Although early-career researchers tend to be the impetus and target for such tools, scientists of all levels can benefit from their use.

In this perspective, we review the status of democratized resource availability and accessibility in bioimaging from the perspective of early-career academic scientists and suggest where improvements can be made to increase utility and discoverability for novice researchers.

## Discussion

### Existing resources that enable widespread availability of microscopy and image analysis training materials

Here, we spotlight existing resources for early-career scientists to assist in their training (see Table 1). These examples of current and democratized resources highlight the range of knowledge necessary for advancement in bioimaging career paths.

**TABLE 1:** Resource table.

Networks and organizations	Link
Global BioImaging	https://globalbioimaging.org/
Global BioImage Analysts’s Society	https://www.globias.org/home
BioImaging North America	https://www.bioimagingnorthamerica.org/
Latin America Bioimaging	https://labi.lat/
Euro-BioImaging	https://www.eurobioimaging.eu/
African BioImaging Consortium	https://www.africanbioimaging.org/
Microscopy Australia	https://micro.org.au/
India BioImaging Consortium	https://www.eurobioimaging.eu/partners/india-bioimaging/
SingaScope	https://www.singascope.sg/
Quality Assessment and Reproducibility for Instruments and Images in Light Microscopy	https://quarep.org/
**Forums**	**Link**
Image.sc	https://forum.image.sc/
Microforum	https://forum.microlist.org/
ITK Forum	https://discourse.itk.org/
KNIME Community Forum	https://forum.knime.com/
Confocal Microscopy Listserv (email subscription based)	https://lists.umn.edu/cgi-bin/wa?SUBED1=confocalmicroscopy&A=1
**Online training material**	**Link**
MicroTutor	https://microtutorcourses.org/
MicroscopyU	https://www.microscopyu.com/
iBiology Techniques	https://www.youtube.com/@iBiologyTechniques
Introduction to Bioimage Analysis	https://bioimagebook.github.io/README.html
Analyzing fluorescence microscopy images with ImageJ	https://petebankhead.gitbooks.io/imagej-intro/content/
Bioimage Analysis Training Resources	https://neubias.github.io/training-resources/all-modules/
**Datasets repositories^a^**	**Link**
Broad Bioimage Benchmark Collection	https://bbbc.broadinstitute.org/
BioImage Archive	https://www.ebi.ac.uk/bioimage-archive/
Cellpose Datasets	https://www.cellpose.org/dataset
Deepcell Datasets	https://datasets.deepcell.org/
Image Data Resource	https://idr.openmicroscopy.org/
**Other helpful resources**	
ImageScience.org	https://imagescience.org/
MicroscopyDB	To add to the databases: https://microscopydb.io/ Access to the content through partner websites
Metrics Reloaded	https://metrics-reloaded.dkfz.de/
BioImage Informatics Index	https://biii.eu/
NFDI4BioImage	https://nfdi4bioimage.github.io/training/

A table showing a list of microscopy and image analysis resources available. Please note that this is not an exhaustive list.

^a^ Check the licensing and terms and conditions before use, as not all repositories are fully open source.

#### Bioimaging societies and organizations provide a network for the community

Bioimaging societies and network organizations leverage digital platforms to facilitate the rapid exchange of ideas and information across geographic locations. These organizations strengthen connections within the field by combining collaboration, professional development, and training opportunities. In addition to providing a network for the bioimaging community, they provide a place to share resources like image informatics tutorials, analysis workshops, financial support, and job postings. Global BioImaging (GBI) (Wright et al., 2024) and the Global BioImage Analysts’ Society (GloBIAS) (Corbat et al., 2025)—cornerstones in the bioimaging community—connect regional bioimaging nodes on a global scale. This regional hierarchy of organizations, including BioImaging North America (BINA) (De Niz et al., 2024) amongst others (Table 1), allows each group to operate within the constraints and goals of their own local communities while remaining connected to the greater international bioimaging network.

Another organization that creates open digital resources for bioimaging scientists is Quality Assessment and Reproducibility for Instruments and Images in Light Microscopy (QUAREP-LiMi) (Nelson et al., 2021). Their goal is to promote best practices in light microscopy and image analysis by encouraging imaging and metadata standardization and by democratizing access to educational materials. Like the aforementioned regional networks, they promote visibility for different tools and events relevant to the bioimaging and image informatics communities. Dedicated and motivated early-career microscopists can join ongoing projects under the mentorship of more experienced users and contribute to writing, image analysis, or code development tasks.

These groups are indispensable to the early-career demographic as they provide a centralized location to learn about bioimaging tools and analysis procedures. Furthermore, many are primarily volunteer-led organizations, providing an avenue for young scientists to actively participate in the larger community. Unlike other scientific societies that require membership fees, the majority of the previously listed bioimaging groups are free to join. Many also assist scientists by offering financial support to those with limited resources. The ability to access these offerings online is another facet that lends itself to easier learning and troubleshooting with imaging and image analysis procedures. Having access to these resource hubs provides a starting point for early-career scientists with limited experience while also fortifying connections between professionals worldwide.

#### Scientific community forums provide direct peer-to-peer and expert-to-peer guidance

Existing online resources play a pivotal role in enabling widespread availability of microscopy and image analysis training materials. Platforms including the Image.sc forum (Rueden et al., 2019), Microforum (Forum, 2025), and Confocal Listserv (List Serv, 2025) provide virtual support to newcomers and experienced professionals alike.

The Image.sc forum fosters a welcoming environment that encourages interactions between newcomers and seasoned image analysts (Sivagurunathan et al., 2024). This is especially advantageous for connecting biological researchers with bioimage analysis experts (Cimini et al., 2024). Novices can post questions and seek advice, while experienced users can find in-depth discussions on topics of interest (Uhlmann et al., 2024). Forum interactions have a widespread impact, often leading to the development of new software and ideas (Song and Goedhart, 2024). Additionally, community partners use this platform to connect with and support their users, enhancing the overall experience. The forum promotes discussions on unifying topics such as metadata and standards and provides centralized support for navigating more than 100 different image file types, many of which are proprietary (Moore et al., 2021). Scientists can turn to Image.sc for assistance with image analysis tasks, which is particularly significant to early-career individuals looking to strengthen their understanding of these strategies. Since its inception by the ImageJ and CellProfiler teams in 2018, Image.sc has grown significantly, boasting 76 community partners and nearly 30,000 members as of March 2025, including numerous active users who provide daily assistance. Its open-access model ensures that relevant discussions are accessible through standard web searches and browser translation tools to reach a global audience. Complementing Image.sc, the Microforum specializes in microscopy hardware, imaging methods, sample preparation, and experimental protocols (Senft et al., 2025). It serves as a dedicated platform for targeted discussions on equipment selection, hardware troubleshooting, and refinement of microscopy techniques. Tangentially, the Confocal Listserv has been a longstanding resource in microscopy communication that facilitates real-time interactions among professionals worldwide (Abrams et al., 2023). This email list shares practical advice, job opportunities, and timely updates on conferences, workshops, and technological advancements. Its global membership helps propagate microscopy-related communications promptly and effectively through a digital medium.

Together, these resources form a comprehensive toolbox, empowering bioimaging scientists at all career stages to access up-to-date, specialized, and high-quality information for advancing their scientific research and professional development through the convenience of the internet.

#### Online training material provides conceptual and practical knowledge

Online training material supports self-paced education for fundamental bioimaging concepts, equipment use, and image analysis. These online guides not only vary in scale but can also vary in media type, including text, video, interactive websites, or live seminars. Educational platforms like MicroscopyU (Nikon MicroscopyU, 2025) and Microtutor (Microtutor Microtutor, 2025) are backed by teams of experts to develop well-structured, comprehensive, and user-friendly resources that have significantly contributed to making microscopy education more accessible. MicroscopyU, launched in 2000 by Nikon and headed by the late Michael W. Davidson, is a knowledge database with interactive tutorials and animations to enhance learning from basic concepts to advanced applications in light microscopy. As this resource becomes outdated, there is a need to create more modern and updated visuals to keep pace with the advancement of the field. Microtutor, launched in 2024 and backed by a 13-person development team, is a free virtual program supported by an online forum to complement learning. Their first course on fluorescence microscopy included a live, online classroom that offered a structured learning experience and real-time interaction with instructors and fellow students.

Other user- and community-created educational content can help to fill in any gaps in learning but are scattered across different online platforms, such as YouTube, GitHub, and core facility websites. One existing solution is MicroscopyDB, which serves as a centralized, searchable database of expert-curated media that can be accessed through a simple web search or the websites of many bioimaging organizations (see Table 1) (Zenner, 2023). Users with microscopy-related resources including jobs, events, or tools can submit them through the MicroscopyDB website to increase community visibility.

#### Publicly accessible datasets offer microscopy data for bioimage analysis training

Early-career individuals may not always have access to reliable image data. This can be the result of a variety of limitations, including financial resources, experience, or access to microscopes and samples. For this reason, publicly available bioimage datasets are valuable, providing raw material for use in training and research that may otherwise be unobtainable.

Several institutes from across the world have taken the initiative to consolidate large imaging datasets for individual use. These collections serve as indispensable tools in the Open Science movement (Elliott and Resnik, 2025), simultaneously promoting wider access to imaging data and enabling the training of well-generalized machine learning models. For example, the popular bioimaging segmentation tool, CellPose, was developed using publicly available datasets, illustrating how open data can lead to advances in image analysis (Stringer et al., 2021). Examples of these datasets have been listed in Table 1 to increase their visibility to early-career scientists looking for imaging data to supplement their work. Such efforts support FAIR (Findability, Accessibility, Interoperability, Reusability) data principles, an important practice in the fields of microscopy and image analysis that also works towards easing the learning curve for early-career users (Wilkinson et al., 2016).

### Limitations of existing resources and challenges to be considered

Though current open-access platforms help level the playing field for bioimaging trainees, obstacles remain that limit the practicality of these tools. Here, we call attention to several challenges of existing resources while providing suggestions to promote their usability. It is our hope that considering and adopting these recommendations for both the improvement of existing tools and the development of new ones will enhance the training of early-career scientists across varying backgrounds and circumstances.

#### Obstacles to resource awareness

Effective application of available tools depends on trainees being aware of their existence. In the introduction, we highlighted the essential services that local core facilities provide (when available) to early-career researchers. Core facilities are also uniquely positioned as frontline champions to promote the plethora of online bioimaging resources that exist, complementing the knowledge of individual supervisors and mentors. However, access to and knowledge of core facilities differs between institutes. Smaller institutes may have more centrally organized core facilities with direct researcher interaction, while the needs of large institutes may require a mix of centralized and departmental organized cores.

We have witnessed examples of core facilities practicing proactive communication within their institute juxtaposed to others with little to no internal engagement. In the latter case, we encourage core faculty to engage with relevant audiences in the form of email listservs, workshops, or talks with an emphasis on trainees and new principal investigators. These connections are stepping stones in the growth of early-career scientists, thereby influencing experimental and professional outcomes. We also encourage knowledgeable individuals to actively share these resources within their networks and institutions to promote them. For example, universities can host core fairs to spotlight the services of shared research facilities to the local community, as done by the Research Core Facility Showcase at the University of Texas at Austin (Gradschool, 2025) and McGill University’s Research Platforms Fest (Healthenews, 2025).

It is also vital that regional bioimaging organizations do their own outreach at smaller scientific meetings and institutions. Presenting in these environments is especially beneficial for trainees that do not possess the resources to travel to large conferences. As previously stated, these networks serve as a centralized hub for comprehensive dissemination of knowledge on the latest bioimaging events, analysis approaches, technologies, and research findings. Thus, the promotion and utilization of these organizations as a resource can open the door to awareness of other community tools as well.

#### Obstacles to language accessibility

Language barriers limit access to tools for early-career scientists in the bioimaging field. English is the universal language of science (Ulrich, 2012), facilitating global communication and collaboration among researchers. However, this universality also means most tools and resources (i.e., informational texts, tutorials, manuals, and code documentation) are only available in English despite the thousands of languages spoken around the world. Thus, increased availability of translation tools and training options in multiple languages would open learning to scientific communities internationally.

One potential solution is leveraging emerging large language models to generate automated documentation, annotations, and translations in multiple languages, helping non-Anglophone scientists gain knowledge in microscopy and image analysis. BINA has begun exploring these technologies to support multilingual accessibility with efforts to facilitate meeting translations (De Niz et al., 2024) and to host events in additional languages. However, the implementation of automated translation tools can be difficult to navigate since microscopy and image analysis are specialized skills that utilize technical vernacular. This terminology can also differ between countries with a shared language, like the varying Spanish dialects spoken in Latin America and Spain, thus necessitating human input for accurate translation. A potential solution would be to develop an open word translation bank for such specialized language, which would help scientists employ auto-translation tools and, in turn, support group translation efforts.

By working to provide freely available translations, we can create a scientific community where people from different linguistic backgrounds can participate and contribute equally. While a common language is important for effective communication in science, creating an open environment goes beyond language proficiency. It involves ensuring that all individuals, no matter their country of origin or native language, feel welcomed and valued within the bioimaging community. This effort will promote innovation and advance scientific knowledge collaboratively across borders and cultures.

#### Obstacles in resource availability

Scientists often conduct innovative research that may not conform to a standard schedule but requires accessing resources with expediency. However, oftentimes these resources do not follow FAIR principles. In some cases, deposited code and standardized datasets are not publicly available for a set period after publication or are only available after making a request to the corresponding author, which hinders their findability and accessibility. This lag creates a bottleneck, which can result in weakened interest in and usage of these resources. Furthermore, depending on the licensing of deposited material, some resources cannot be reused. We encourage scientists and investigators to adopt FAIR principles, to ensure that code and sample data are readily downloadable at the time of publication or announcement, and to carefully consider copyright and licensing restrictions (Haase, 2025). Researchers should consider using repositories, for example, GitHub for code and BioImage Archive for datasets, to make material more available. Furthermore, we challenge developers to make the entirety of their resources available on-demand, to be utilized by early-career scientists and the broader scientific community.

Knowledge dissemination through live courses and seminars has experienced a major shift from a focus on in-person learning to more globally accessible self-paced training. While platforms such as Zoom and Webex have significantly enhanced access to scientific discourse, some barriers such as time zone differences and unreliable internet access can limit engagement for many aspiring researchers.

To address these challenges, we encourage that seminars and scientific discussions be recorded and made available as downloadable material or on public media platforms, ensuring unrestricted access to educational resources. Furthermore, virtual conferences that adopt asynchronous participation models can incorporate scheduled sessions across multiple time zones to maximize availability. One successful execution of this strategy is GBI’s Career Path Spotlight Series, which is hosted at two different times to accommodate both the Eastern and Western hemispheres (Wright et al., 2024; Globalbioimaging, 2025). The Virtual I2K Conference has further exemplified the success of asynchronous virtual conferences by providing a calendar with live updates throughout the event and offering flexibility for both presenters and attendees (Virtual I2K 2024, 2025). Conference videos are then made publicly available online, further enabling image informatics access as needed. These efforts have facilitated the growth of the global bioimaging community, demonstrating that when resources are made universally accessible, the scientific community flourishes. Moving forward, greater adoption of adaptive digital infrastructure, creating downloadable educational content, and hybrid or asynchronous participation models will foster a more democratized bioimaging community.

#### Obstacles in learning with limited prior knowledge

Many bioimaging tools and resources are available online for early-career scientists, yet access to these foundational knowledge resources remains limited. For those entering the bioimaging field, understanding core microscopy and image analysis concepts (i.e., signal detection, image acquisition settings, file formats) is essential. However, without proper orientation, it may be difficult to navigate these concepts—how does a novice find the tutorial needed if they do not know what resource to look for in the first place? To yield the best results, trainees should consult with expert bioimage analysts as these professionals provide a unique skillset when extracting information from image datasets (Cimini et al., 2024).

Training programs necessarily assume a certain extent of prior knowledge, which can often leave beginners without the support they need at the entry level. One particular gap is the lack of fundamental training in image analysis when starting out in microscopy. Many early-career scientists struggle with processing and analyzing the images they acquire. Open-source software like FIJI/ImageJ (Schindelin et al., 2012), Napari (Sofroniew et al., 2025), QuPath (Bankhead et al., 2017), and their plugins, provide essential functionalities for image processing but often lack clear, user-friendly documentation and structured tutorials, making it even more challenging for beginners to learn independently. The vast majority of these tools are also only available in English, compounding the learning curve for non-Anglophones. We call upon developers to keep trainees in mind when providing sufficient documentation and tutorials for their software.

A notable initiative for broadening access to bioimaging among non-specialized audiences is the Mexican Bioimaging Workshops, launched in 2022 (De Niz et al., 2024). This effort has organized in-person and online courses covering topics from fundamental imaging and analysis concepts like Köehler illumination and automating image analysis, to advanced techniques like light sheet and super-resolution microscopy. This program has made bioimaging possible for individuals who would not otherwise have access to this level of education. Expanding upon these resources will allow early-career scientists worldwide to build a strong microscopy knowledge foundation before advancing in their specialization of choice.

## Conclusion

Although breaking into the bioimaging field can be challenging, the microscopy and image informatics community has developed a range of accessible tools to help early-career scientists overcome limitations associated with resources and expertise. Bioimaging scientists at all career stages benefit from openly available datasets for practicing analysis methods, online forums for image informatics guidance, and professional development through community networks. Obstacles in accessibility persist despite these advancements, highlighting the need for both community involvement and institutional support. A large component of the solution to accessibility issues lies within their digital access. Given these challenges, the bioimaging community must remain dedicated to promoting democratized education and working to ensure that best microscopy practices are universal.

## Data Availability

The original contributions presented in the study are included in the article/supplementary material, further inquiries can be directed to the corresponding author.

## References

[B1] Abrams B. Pengo T. Wee T.-L. Deagle R. C. Vuillemin N. Callahan L. M. (2023). Tissue-like 3D standard and protocols for microscope quality management. Microsc. Microanal. 29 (2), 616–634. 10.1093/micmic/ozad014 37749742 PMC10617369

[B2] Bankhead P. Loughrey M. B. Fernández J. A. Dombrowski Y. McArt D. G. Dunne P. D. (2017). QuPath: open source software for digital pathology image analysis. Sci. Rep. 7 (1), 16878. 10.1038/s41598-017-17204-5 29203879 PMC5715110

[B3] Cimini B. A. Bankhead P. D'Antuono R. Fazeli E. Fernandez-Rodriguez J. Fuster-Barceló C. (2024). The crucial role of bioimage analysts in scientific research and publication. J. Cell Sci. 137 (20), jcs262322. 10.1242/jcs.262322 39475207 PMC11698046

[B4] Corbat A. A. Walther C. G. de la Ballina L. R. Condon N. D. Felder A. A. Schätz M. (2025). GloBIAS: strengthening the foundations of BioImage analysis. LID - arXiv:2507.06407v1. 2331–8422. 10.1038/s41592-026-03060-7 42020774

[B5] De Niz M. Escobedo García R. Terán Ramirez C. Pakowski Y. Abonza Y. Bialy N. (2024). Building momentum through networks: bioimaging across the americas. J. Microsc. 294 (3), 420–439. 10.1111/jmi.13318 38747464 PMC11152921

[B6] Elliott K. C. Resnik D. B. (2025). Making open science work for science and society, 1552–9924. 10.1289/EHP4808 PMC6792383 31353949

[B7] Forum (2025). Microforum microlist. Available online at: https://forum.microlist.org/ (Accessed April 18, 2025).

[B8] Globalbioimaging (2025). Spotlight seminar Series global BioImaging. Available online at: https://globalbioimaging.org/working-groups/career-paths/spotlight-seminar-series.

[B9] Gradschool (2025). Research core facility Showcase. Austin, TX: University of Texas at Austin. Available online at: https://gradschool.utexas.edu/events/research-core-facility-showcase.

[B10] Haase R. (2025). FocalPlane2023. Available online at: https://focalplane.biologists.com/2023/05/06/if-you-license-it-itll-be-harder-to-steal-it-why-we-should-license-our-work/.

[B11] Healthenews (2025). First research platforms fest put spotlight on McGill’s world-class core Platforms1 april 2025. Available online at: https://healthenews.mcgill.ca/first-research-platforms-fest-put-spotlight-on-mcgills-world-class-core-platforms/.

[B12] List Serv (2025). Confocal microscopy list Serv. Available online at: https://lists.umn.edu/cgi-bin/wa?A0=confocalmicroscopy.

[B13] Microtutor Microtutor (2025). Microtutor microtutor. Available online at: https://microtutorcourses.org/ (Accessed April 18, 2025).

[B14] Moore J. Allan C. Besson S. Burel J.-M. Diel E. Gault D. (2021). OME-NGFF: a next-generation file format for expanding bioimaging data-access strategies. Nat. Methods 18 (12), 1496–1498. 10.1038/s41592-021-01326-w 34845388 PMC8648559

[B15] Nelson G. Boehm U. Bagley S. Bajcsy P. Bischof J. Brown C. M. (2021). QUAREP-LiMi: a community-driven initiative to establish guidelines for quality assessment and reproducibility for instruments and images in light microscopy. J. Microsc. 284 (1), 56–73. 10.1111/jmi.13041 34214188 PMC10388377

[B16] Nikon MicroscopyU (2025). The source for microscopy education MicroscopyU. Available online at: https://www.microscopyu.com/.

[B17] Rueden C. T. Ackerman J. Arena E. T. Eglinger J. Cimini B. A. Goodman A. (2019). Scientific Community Image Forum: a discussion forum for scientific image software. PLoS Biol. 17 (6), e3000340. 10.1371/journal.pbio.3000340 31216269 PMC6602289

[B18] Schindelin J. Arganda-Carreras I. Frise E. Kaynig V. Longair M. Pietzsch T. (2012). Fiji: an open-source platform for biological-image analysis. Nat. Methods 9 (7), 676–682. 10.1038/nmeth.2019 22743772 PMC3855844

[B19] Senft R. A.-O. Diaz-Rohrer B. Colarusso P. Swift L. Jamali N. Jambor H. (2025). A biologist's guide to planning and performing quantitative bioimaging experiments, 1545–7885. 10.1371/journal.pbio.3002167 PMC10298797 37368874

[B20] Sivagurunathan S. Marcotti S. Nelson C. J. Jones M. L. Barry D. J. Slater T. J. A. (2024). Bridging imaging users to imaging analysis – a community survey. J. Microsc. 296 (3), 199–213. 10.1111/jmi.13229 37727897 PMC10950841

[B21] Sofroniew N. Lambert T. Bokota G. Nunez-Iglesias J. Sobolewski P. Sweet A. (2025). napari: a multi-dimensional image viewer for Python (v0.6.0a1). Zenodo. 10.5281/zenodo.15193038

[B22] Song X. Goedhart J. (2024). EzReverse – a web application for background adjustment of color images. bioRxiv. 2024. 10.1101/2024.05.27.594095

[B23] Stringer C. Wang T. Michaelos M. Pachitariu M. (2021). Cellpose: a generalist algorithm for cellular segmentation. Nat. Methods 18 (1), 100–106. 10.1038/s41592-020-01018-x 33318659

[B24] Uhlmann V. Hartley M. Moore J. Weisbart E. Zaritsky A. (2024). Making the most of bioimaging data through interdisciplinary interactions. J. Cell Sci. 137 (20), jcs262139. 10.1242/jcs.262139 39440474 PMC11529881

[B25] Ulrich A. (2012). Linguistic inequality and its effects on participation in scientific discourse and on global knowledge accumulation – with a closer look at the problems of the second-rank language communities. Appl. Linguist. Rev. 3(2):333–355. 10.1515/applirev-2012-0016

[B26] Virtual I2K 2024 (2025). Free online tutorials on image analysis from october 28-30, 2024 I2K 2024. Available online at: https://www.i2kconference.org/virtual.html.

[B27] Wilkinson M. D. Dumontier M. Aalbersberg I. J. Appleton G. Axton M. Baak A. (2016). The FAIR Guiding Principles for scientific data management and stewardship. Sci. Data 3 (1), 160018. 10.1038/sdata.2016.18 26978244 PMC4792175

[B28] Wright G. D. Thompson K. A. Reis Y. Bischof J. Hockberger P. E. Itano M. S. (2024). Recognising the importance and impact of Imaging Scientists: global guidelines for establishing career paths within core facilities. J. Microsc. 294 (3), 397–410. 10.1111/jmi.13307 38691400

[B29] Zenner H. L. (2023). Coming into focus. J. Cell Sci. 136 (24), jcs261840. 10.1242/jcs.261840 38158845

